# Dances with doves, hawks and eagles: Realising the potential of emotion during simulation

**DOI:** 10.1111/medu.15588

**Published:** 2024-12-04

**Authors:** Russell Peek

**Affiliations:** ^1^ Three Counties Medical School University of Worcester Worcester UK; ^2^ Gloucestershire Hospitals NHS Foundation Trust Gloucester UK

## Abstract

.@russell_peek argues that simulation‐based education offers an opportunity to enhance learning because it evokes an emotional response in learners, rather than despite it doing so.

The Learner approached the steep white steps leading down to the simulation suite, hastily scribbled notes under her arm, her mind alive with the task ahead. As she pushed through the door, she was greeted by the familiar sights and sounds of the almost‐clinical space. The Learner offered a hesitant smile to her fellow students and silently rehearsed the simulation mantra, planning her strategy as this week's team leader. Her mind wandered to who would be facilitating the session. Would it be the Dove, supportive and kind, ready to extend an olive branch if things got out of hand? Or the Hawk, intense and expectant, introducing unanticipated twists and turns if it all seemed to be going too well. Perhaps it would be the Eagle, steadfast and reliable, inscrutable until it was all done bar the talking. In the end, did it really matter? She would get through it, and there would be time to reflect on what was, and what could have been. A, B, C, D, E …

There is accumulating evidence that Simulation‐Based Education (SBE) evokes emotional responses in learners, and that these responses can affect performance and educational outcomes.^1^ In this issue of Medical Education, Behrens et al. explore how simulation facilitators perceive students' emotions and react to them during SBE.^2^ Facilitators reported that they recognise emotional states through verbal and non‐verbal cues, including patterns of speech, facial expressions and body language. Emotional states were identified as ‘positive’ (e.g. smiling, relaxed body posture, confident speech) or ‘negative’ (e.g. frowning, tense posture, nervousness). The authors identify and describe three patterns of facilitator response to perceived emotion: managing simulation complexity to reduce negative emotions; ‘turning up the heat’ to push students in preparation for real‐world practice; or sticking to the script for consistency, addressing emotions only in debriefing. Each pattern of response was rooted in facilitators' desire to optimise learning from SBE, with an individual's approach influenced by their personal educational philosophy, beliefs and experience.

Simulation‐Based Education (SBE) evokes emotional responses in learners, and these responses can affect performance and educational outcomes.

This interpersonal variation suggests that educators hold a range of beliefs about how emotion affects learning in SBE, and whether or not we should intervene when observed behaviour suggests an emotional response in participants. It also highlights the complexity of relationships between experience, emotion, behaviour and outcomes. Theoretical frameworks aiming to untangle these relationships describe physiological, emotional and behavioural responses to largely subconscious processes as we interact with our environment. For example, the biopsychosocial model of challenge and threat proposes that physiological stress responses are driven by appraisal of situational demands and personal resources.^3^ Challenge, experienced when resources are perceived to meet or exceed demands, is associated with more adaptive physiological responses and improved performance compared to threat, experienced when demands exceed resources. Control‐value theory (CVT) proposes that positive or negative emotions are triggered by perceived ability to influence outcomes (control appraisals) and the importance or relevance of a task (value appraisals).^4^ Both positive and negative emotions can be activating (thereby motivating or enhancing engagement) or deactivating (demotivating or impairing performance). Such theoretical frameworks point to the complex and largely hidden internal processes linking experience to emotional and behavioural responses. As educators, we should perhaps exercise caution in assuming we can reliably identify emotional states and their drivers by simply observing learners.

Educators hold a range of beliefs about how emotion affects learning in SBE, and whether or not we should intervene when observed behaviour suggests an emotional response in participants.

There are additional challenges for facilitators seeking to respond optimally to learners' emotional states during SBE, beyond accurately identifying and interpreting verbal or behavioural cues. Positive and negative emotions can coexist, the intensity of emotional arousal varies within and between learners, and responses may differ between simulated and real‐world experiences.^3^ In the face of so much uncertainty, it is easy to understand why facilitators adopt different approaches to a common goal. However, despite the challenges, there is an opportunity to enhance learning through SBE *because* it evokes an emotional response, rather than *despite* it doing so.

A little over 30 years ago, emotional intelligence (EI) was proposed as a psychological construct analogous to general intelligence but characterised by a set of abilities to perceive, understand, use and manage emotions in oneself and others, requiring self‐awareness, self‐regulation, empathy and interpersonal skills.^5^ Despite empirical challenges in demonstrating EI as a distinct construct and in developing reliable measurement tools, it has percolated into popular culture and is widely regarded as beneficial for job satisfaction, work performance, effective leadership and personal well‐being.^6^ There are parallels between the abilities underpinning emotional intelligence and elements of medical professionalism, and indeed EI has been linked with physical and emotional caring behaviours, clinical decision making and enhanced patient trust, satisfaction and patient–clinician relationships.^7^, ^8^, ^9^

Beyond clinical practice, emotionally intelligent educators might be expected to be more accurate in their perception of learners' emotional states, to understand the emotional impact of SBE and to be student‐centred when considering how best to use and manage emotion in simulation design and facilitation. SBE offers the opportunity to explicitly explore emotional responses, developing self‐awareness in learners and allowing facilitators to check the validity of their perceptions and expectations. The study of emotion in SBE is in its infancy and tends to focus on negative emotions, such as fear or anxiety.^10^ However, SBE could help learners develop strategies to manage and use the emotional response to challenging situations, for example, through arousal reappraisal.

SBE could help learners develop strategies to manage and use the emotional response to challenging situations, for example, through arousal reappraisal.

Intervention to break the association between physiological arousal and a negative interpretation can reduce anxiety and improve performance under pressure.^11^, ^12^ Learning to reframe the physiological arousal experienced in SBE as positive may increase the likelihood of an emotional response being felt as beneficial (activating) rather than debilitating (deactivating).

Intervention to break the association between physiological arousal and a negative interpretation can reduce anxiety and improve performance under pressure.

Behrens et al.'s eloquent description of ways in which SBE facilitators recognise and respond to perceived emotional states suggests that educators are sensitive to the emotional impact of SBE and wish to maximise the value of SBE for learners. This should include actively considering the experience of emotion as a learning opportunity in itself. Our challenge now is to explore whether the emotionally intelligent thing to do is to dance with emotions like the dove, the hawk or the eagle.

## AUTHOR CONTRIBUTIONS

**Russell Peek:** Writing – original draft.
